# The Impact of the COVID-19 Pandemic on People With Lymphedema in an Endemic Area for Lymphatic Filariasis in Brazil

**DOI:** 10.3389/ijph.2023.1605317

**Published:** 2023-01-12

**Authors:** Lígia Tomaz de Aquino, Ana Maria Aguiar-Santos, Abraham Rocha, Artur Vinicius de Araujo Coutinho, Mirella Silva Batista do Nascimento, Eduarda Correia Moretti, Fernando Leonel da Silva, Fábia Maria Lima, Cristine Bonfim, Zulma Medeiros

**Affiliations:** ^1^ Postgraduate Health Sciences, University of Pernambuco, Recife, Brazil; ^2^ National Filarial Reference Service, Department of Parasitology, Aggeu Magalhães Institute, Oswaldo Cruz Foundation, Recife, Brazil; ^3^ Physiotherapy Department, Recife’s Estacio University Center, Recife, Brazil; ^4^ Institute of Biological and Health Sciences, Federal University of Alagoas, Maceió, Brazil; ^5^ Nursing School, University of Pernambuco, Recife, Brazil; ^6^ Postgraduate Program in Public Health, Federal University of Pernambuco, Recife, Brazil; ^7^ Social Research Department, Joaquim Nabuco Foundation, Recife, Brazil

**Keywords:** COVID-19, lymphatic filariasis, lymphedema, morbidity, quality of life

## Abstract

**Objectives:** To investigate the repercussions of the COVID-19 pandemic on lymphedema patients from an endemic area of lymphatic filariasis.

**Methods:** The study descriptive compared sociodemographic and clinical aspects, risk of falling and quality of life, prior and during the COVID-19 pandemic in 28 lymphedema patients, older than 18 years old and under investigation of filarial infection. For the evaluation of functional mobility, the *Time Up and Go* test and *The Medical Outcome Study Short Form-36 Health* for quality of life, was used.

**Results:** An increase in interdigital and dermal lesions, a higher frequency of acute dermatolymphangioadenitis crises and risk of falling, worsening of quality of life in the domains of physical functioning, general health, vitality, and mental health during the pandemic was observed.

**Conclusion:** Our findings of clinical worsening and quality of life of patients during the COVID-19 pandemic indicate the need to reinforce the goal of the Lymphatic Filariasis Program regarding the follow-up of these patients in the actions of the Global Program for the Elimination of Lymphatic Filariasis, due to the discontinuity in the care during the pandemic.

## Introduction

Lymphatic filariasis is a neglected tropical disease and, in Brazil, its etiologic agent is the parasite *Wuchereria bancrofti* and has *Culex quinquefasciatus* as the only vector (1). In Brazil, the state of Pernambuco, northeast of the country, is considered the last endemic area, and is represented by the municipalities of Recife, Olinda, Jaboatão dos Guararapes and Paulista. Annual mass drug administration (MDA) with isolated diethylcarbamazine was implemented in 2003–2017 in three of these endemic municipalities, and in Paulista, due to its low endemicity control, actions have been restricted to the individual treatment of detected cases of microfilaremia. Pernambuco has conducted the Transmission Assessment Survey (TAS) following the cessation of MDA with verification of the interruption of transmission. These data will compile a dossier seeking validation of the elimination of LF from the World Health Organization (2). Adult worms cause damage to the lymphatic system with consequent stasis of lymph in the tissues and may progress to one of its main clinical manifestations: lymphedema or its more advanced condition, elephantiasis, a serious, progressive, chronic and disabling condition. Patients with lymphedema have physical limitations, frequent comorbidities, and consequent socioeconomic problems, in addition to feelings of embarrassment and emotional distress. Therefore, filarial morbidity is considered a public health problem (3).

In 2000, the World Health Organization (WHO) launched the Global Program to Eliminate Lymphatic Filariasis (GPELF) with a view to eliminating it as a public health problem with goals for 2030. The GPELF has two fundamental pillars: breaking transmission through mass drug administration (MDA) and managing morbidity and preventing disability (MMPD) (4). To reach the second pillar, as they are chronic and often irreversible manifestations, the focus is to alleviate and reduce the suffering caused by hydrocele and lymphedema. The episodes of secondary infections, acute dermatolymphangioadenitis (ADLA) are the most associated and debilitating complications with lymphedema and elephantiasis (5–7).

In order to alleviate and reduce the suffering among people with lymphedema and to obtain an improvement in the quality of life and prevention of disabilities, prophylaxis and treatment of ADLA episodes are recommended. For such, simple and continuous measures are indicated, which include hygiene and skin care, effective treatment of skin lesions, elevation of the affected limb, use of adequate footwear and, ideally, physiotherapy (5–9). In addition to these measures, and to improve the quality of life and reduce the suffering of patients with morbidity, the WHO recommends a basic care package, with several items, which include continued access to the basic health system (10).

From 2019, with the COVID-19 pandemic, the main actions taken to prevent this viral infection were individual hygiene measures, social distancing and later, when available, vaccination (11–13). The pandemic had a great impact in the whole world, in addition to high mortality, which resulted in negative and indirect effects on other diseases (14, 15). Many health programs have been discontinued and the consequences of these cessations are still completely unknown. The GPELF was also impacted by the interruption of the MDA and stoppage of all activities and the possible delay in meeting the elimination targets, such as public health planned for 2030 (16).

The delay in fulfilling the elimination of transmission may cause higher morbidity levels while transmission is ongoing. Mitigation and acceleration strategies are being considered to prevent further damage to filariasis control, such as: increase of MDA coverage to 80%, implementing treatment twice a year and, when possible, triple association of antifilarial drugs with ivermectin, diethylcarbamazine and albendazole (16). Even with these measures, the sharp rise in poverty associated with COVID-19 is a worrying factor and could derailPGELF’s promising efforts (17). Filarial morbidity management requires continuous patient follow-ups, particularly those with lymphedema, to obtain adequate management and improve quality of life (7). It is necessary to investigate the impact that the COVID-19 pandemic has had on people with lower limb lymphedema residing in an endemic area of lymphatic filariasis in Pernambuco, in the northeast of Brazil, through comparison of data obtained in 2019 and 2021, respectively before and during the pandemic.

## Methods

### Study Design and Sampling

The study design was descriptive, and data were collected in the second half of 2019 and 2021, prior and during the COVID-19 pandemic, respectively. Twenty-eight patients were included in follow-up care at the outpatient clinic of the National Reference Service for Filariasis at the Oswaldo Cruz Foundation in Pernambuco - Brazil. This service is the only one in the country, linked to the health public system, the Sistema Único de Saúde (SUS), which ranges from primary care to complex procedures, that provides multidisciplinary assistance in the investigation and follow-up of patients with morbidity from an endemic area of lymphatic filariasis (18).

The patients included had a diagnosis of lymphedema of the lower limbs, were older than 18 years old, had no active infection by *W. bancrofti*, and were assessed for functional mobility and quality of life. All participants were investigated using circulating microfilariae and quantified using the polycarbonate membrane filtration technique with 3-mm pores (Nucleopore^®^) (19) and by searching for the ‘filarial dance sign’ using ultrasound (20). In order to test for the circulating filarial antigen for *W. bancrofti*, the point-of-care immunochromatographic test-AD12 (POC-ICT-AD12 - Alere, Inc., Scarborough, United States) was used. According to the manufacturer’s instructions, the enzyme-linked immunosorbent assay—Og4C3-ELISA (TropBio^®^ Pty Ltd., Townsville, Queensland, Australia) (21) and Alere TM Filariasis test strips were used (22).

### Research Protocol

Information regarding the period before the pandemic was collected from medical records and data related to the pandemic period through interviews, clinical examination and application of specific tests. In the sociodemographic evaluation, information related to sex, age, marital status, literacy, occupation, housing, number of people in the household and monthly family income.

Information regarding clinical aspects were the number of acute attacks (ADLA), stages of lymphedema, maintenance of self-care and hygiene of the affected limb, ulcers or skin surface lesions, interdigital lesions as well as access to continuous use drugs and form of its acquisition. Vaccination coverage and infection for COVID-19 were also recorded.

Clinical examination was performed to evaluate lymphedema, identifying the edema as unilateral or bilateral and stages classified according to the criteria of Dreyer (23). Perimetry was measured at ten points on the lower limb, taking the apex of the patella as a reference (zero point), with four measurements every 7 cm above, and four measurements below, in addition to the malleolar point (8).

*Time Up and Go (TUG*) test was used to evaluate the functional mobility of the lower limbs. In performing the *TUG*, the individual was instructed to get up from a standardized chair and, after the verbal command, to walk 3 m, turn around, walk back to the chair, and sit down. The patient was instructed to walk at a fast, comfortable and safe pace, without receiving any physical assistance and not talking during the test. All guidelines were previously provided, and three collections were taken for the test, with intervals of 20 s between them. The timer was started with the first anterior movement of the body and ended when the patient sat in the chair and supported his/her back. The arithmetic mean of the time values, in seconds, obtained in the three collections was considered the result of the *TUG*. The reference values for the risks of fall were low <10 s; moderate between 10–19 s and high ≥20 s (24, 25).

The *Medical Outcome Study Short* Form*-36 Health Survey* (SF-36) was used to assess quality of life with 36 questions and eight domains (Physical functioning, Limitations of physical health, Bodily pain, General health, Vitality, Social functioning, Limitations of emotional problems and Mental health) that generally assess the perception of the disease from the patient’s point of view (26). In the assessment of quality of life, the domains range from zero to one hundred (0–100), where zero represents the worst situation and 100 would be the best for each domain. As it is the most adopted instrument in studies worldwide, recommended by the World Health Organization (WHO), it is considered the gold standard in assessing quality of life (26).

### Data Analysis

Initially, the relative and absolute frequencies of the categorical variables and the median and interquartile range for the numerical variables were computed.

To identify possible changes in the variables under analysis between the period of the COVID-19 pandemic and the previous period, the following statistical tests were applied: Fisher’s exact test for categorical variables and Wilcoxon test (non-parametric test equivalent to the t-test for data paired) for numeric variables. All calculations were made using the R language version 4.1.0. The significance level of the study was set at 5%.

### Ethical Consideration

This project obtained ethical certification from the Ethics Committee of the University of Pernambuco (protocol number 45608721.1.0000.5192). All participants signed an information and consent form.

## Results

No patient presented active filarial infection at the time of this research. Table 1 shows the profile of the 28 individuals with the following predominant characteristics: 71.4% (*n* = 20) were female; 60.7% (*n* = 17) were 60 years of age or younger, where the median age was 56 years with an interquartile range of 51.8–69 years; 42.9% (*n* = 12) were married or in a stable relationship; 96.4% (*n* = 27) literate; 89.3% (*n* = 25) had their own house; 67.9% (*n* = 19) practiced self-care and hygiene of the lymphoedema limb; all were vaccinated for COVID-19 and 57.1% (*n* = 16) used the Aztrazeneca vaccine in the first 2 doses. They were all vaccinated for COVID-19 with the two doses and two participants had the infection but did not progress to death.

**TABLE 1:** Sociodemographic and clinical characteristics of patients with lymphoedema from an endemic area for lymphatic filariasis (Pernambuco, Brazil, 2019–2021).

Variables	Total patients N = 28 (%)	95% CI
Age^a^	56 (51.8, 69)	
Sex		
Female	20 (71.4%)	51–86
Male	8 (28.6%)	14%–49%
Marital status		
Married/stable union	12 (42.9%)	25%, 63%
Separated/divorced	6 (21.4%)	9.0%, 41%
Single	3 (10.7%)	2.8%, 29%
Widowed	7 (25%)	11%, 45%
Literate		
Yes	27 (96.4%)	80%, 100%
No	1 (3.6%)	0.19%, 20%
Housing		
Owner	25 (89.3%)	71%, 97%
Rented	3 (10.7%)	2.8%, 29%
Maintenance of self-care and hygiene with the affected limb		
Yes	19 (67.9%)	48%, 83%
No	9 (32.1%)	17%, 52%
COVID-19 vaccine (02 doses)		
Astrazenica	16 (57.1%)	37%, 75%
Coronavac	12 (42.9%)	25%, 63%

^a^Median (IQR-Interquartile Range).

Regarding the verification of changes in sociodemographic and clinical characteristics before and during the COVID-19 pandemic (Table 2), there are the following significant findings: the proportion of patients with family income above 1 minimum wage increased by 3.6 times (*p* = 0.026); Access to medicines, which before the pandemic was exclusively done through the SUS, during the COVID-19 pandemic presented other means, especially SUS + Purchased + Others with 57.1% (*p* < 0.001); the probability of having interdigital lesions (*p* < 0.001) and ulcers/dermal lesions (*p* = 0.02) increased by 7 and 5-fold, respectively, during COVID-19.

**TABLE 2:** Distribution of sociodemographic and clinical characteristics prior and during the COVID-19 pandemic in patients with lymphedema from an endemic area for lymphatic filariasis (Pernambuco, Brazil, 2019–2021).

Variables	Prior COVID-19 N, %	During COVID-19 N, %	Difference^a^ (95% CI)	*p*-value*
Occupation				
Free-lance service	4 (14.3%)	3 (10.7%)	—	0.709
Retired	18 (64.3%)	21 (75%)	—	
Unemployed	6 (21.4%)	4 (14.3%)	—	
Montly family income^b^				
≤1 national minimum wage^b^	25 (89.3%)	16 (61.5%)	—	0.026
2–3 national minimum wage^b^	3 (10.7%)	10 (38.5%)	—	
Access to drugs of continuous use				<0.001
SUS^c^	28 (100%)	5 (17.9%)	—	
Purchased	—	3 (10.7%)	—	
SUS + Purchased + Others	—	16 (57.1%)	—	
SUS + Others	—	4 (14.3%)	—	
Stages of lymphoedema				0.77
Stage 2	12 (42.9%)	9 (32.1%)	—	
Stage 3	11 (39.3%)	12 (42.9%)	—	
Stage 4	—	1 (3.6%)	—	
Stage 5	5 (17.9%)	6 (21.4%)	—	
Location of lymphedema				0.11
Unilateral	18 (64.3%)	11 (39.3%)	—	
Bilateral	10 (35.7%)	17 (60.7%)	—	
Interdigital lesions				<0.001
Yes	6 (21.4%)	25 (89.3%)	—	
No	22 (78.6%)	3 (10.7%)	—	
Ulcers or skin surface lessions				0.02
Yes	18 (64.3%)	26 (92.9%)	—	
No	10 (35.7%)	2 (7.1%)	—	
Number of people in the household	2 (2, 3)^f^	3 (3, 4)^f^	−1.50 (−1.99, −0.50)	0.001
ADLA crises^d^	1 (1, 1)^f^	2 (2, 3)^f^	−1.49 (−1.5, −1.0)	<0.001
TUG^e^	8 (6.3, 9)^f^	14.9 (10.5–21.4)^f^	−8.09 (−9.75, −4.94)	<0.001
Perimetry				
Points of leg circumference (28)	61.5 (55.5, 69)^f^	69.8 (61.6–76.4)^f^	−6.75 (−7.75, −6.25)	<0.001
Points of leg circumference (21)	58.8 (49.9, 65.2)^f^	66.8 (56.3, 72.3)^f^	−6.25 (−7.50, −5.25)	<0.001
Points of leg circumference (14)	53 (46, 61)^f^	60.2 (52.1, 64.8)^f^	−5.75 (−6.50, −4.75)	<0.001
Points of leg circumference (07)	49.8 (43, 55.2)^f^	54 (49.2, 62)^f^	−5.99 (−6.90, −4.75)	<0.001
Points of leg circumference (00)	41.8 (38.9, 49.7)^f^	48.8 (45.4, 57.9)^f^	−6.75 (−8.00, −5.75)	<0.001
Points of leg circumference (07-b)	44.5 (36.4, 52.2)^f^	50.8 (45, 58.4)^f^	−5.90 (−7.99, −4.85)	<0.001
Points of leg circumference (14-b)	44 (36.9, 55.2)^f^	52.5 (46, 60.5)^f^	−6.90 (−8.35, −5.75)	<0.001
Points of leg circumference (21-b)	40.5 (31, 50)^f^	49.5 (41.8, 57.1)^f^	−7.25 (−9.99, −5.99)	<0.001
Points of leg circumference (28-b)	36.2 (28.5, 43.4)^f^	42.8 (38, 56.6)^f^	−8.75 (−11.3, −6.50)	<0.001
Points of leg circumference (Maleolar)	28.8 (25.8, 35.1)^f^	36 (31.8, 48.9)^f^	−8.30 (−10.75, −6.35)	<0.001

*Fisher or Wilcoxon tests.

^a^ The Wilcoxon test statistic was used to calculate the difference between the medians. Since pairing is considered, the values may differ from the difference between the presented medians.

^b^ Brazilian minimum wage = US$ 225.86.

^c^ Sistema Único de Saúde (SUS)—Free medicine from public health service in Brazil.

^d^ ADLA, Acute Dermatolymphangioadenitis.

^e^ TUG, time up and go test.

^f^ Median (IQR-Interquartile Range).

During the COVID-19 pandemic, the median of numerical variables showed the following significant changes: the number of people living in the same environment increased by 50% (*p* = 0.001); The number of ADLA crises doubled (*p* < 0.001); the time evaluated in the TUG test increased by about 86% (*p* < 0.001); All points of the evaluated leg circumference had an increase of approximately 16%, where the greatest increase was 22% found at the malleolar point, and the smallest, at point 7 with 8.4% (all with *p* < 0.001).

Table 3 shows that in patients with lymphedema, the quality of life in the eight domains analyzed was bad before and during the pandemic. The previously low scores worsened during the pandemic in the bodily pain domains (19.2 vs. 18.6; *p* = 0.002) and social functioning (24.0 vs. 20.8 *p* < 0.001). However, the domains physical functioning (49.0 vs. 49.5; *p* < 0.001), general health (24.1 vs. 24.5; *p* < 0.001), vitality (19.1 vs. 19.6; *p* = 0.006) and mental health (19.1 vs. 19.3; *p* < 0.001) had slightly higher scores during the pandemic. There was an improvement in limitations due to emotional problems during the pandemic (98.3 vs. 92.3; *p* < 0.001).

**TABLE 3:** Variation in medical outcome study short form-36 health survey in 28 patients with lymphedema from an endemic area for lymphatic filariasis prior and during the COVID pandemic (Pernambuco, Brazil, 2019–2021).

Variables	Prior COVID-19 median (IQR)	During COVID-19 median (IQR)	Difference^a^ (95% CI)	*p*-value*
Physical functioning	49.0 (48.8, 49.4)	49.5 (49.4, 49.5)	−0.50 (−0.675, −0.225)	<0.001
Limitations of physical health	98.5 (98.0, 98.8)	99.0 (99.0, 99.0)	−0.50 (−0.75, 2.99)	0.321
Bodily Pain	19.2 (18.8, 19.5)	18.6 (18.6, 18.8)	0.389 (0.195, 0.559)	0.002
General health	24.1 (24.0, 24.2)	24.5 (24.5, 24.5)	−0.37 (−0.42, −0.32)	<0.001
Vitality	19.1 (19.1, 19.2)	19.6 (19.5, 19.6)	−0.325 (−0.45, −0.15)	0.006
Social functioning	24.0 (23.8, 24.4)	20.8 (20.8, 21.4)	3.00 (1.575, 3.20)	<0.001
Limitations of emotional problems	98.3 (98.0, 99.0)	92.3 (92.3, 93.1)	4.99 (4.99, 5.99)	<0.001
Mental health	19.1 (19.0, 19.2)	19.3 (19.3, 19.3)	−0.20 (−0.24, −0.14)	<0.001

IQR, interquartile range; SF-36, Medical Outcome Study Short Form-36 Health Survey.

*Pairet t or **Wilcoxon tests.

^a^ The Wilcoxon test statistic was used to calculate the difference between the medians. Since pairing is considered, the values may differ from the difference between the presented medians.

## Discussion

The study has shown information concerning social, clinical and quality of life issues among lymphedema patients residing in an endemic area of filariasis during the COVID-19 pandemic. There was an increase in interdigital and dermal lesions/ulcers causing a higher frequency of ADLA crises and the risk of fall, worsening of quality of life in the domains of bodily pain and social functioning.

Most lymphedema cases in females reinforces studies carried out in other endemic areas such as Haiti (27) and Ghana (28). Some findings were surprising for the moment, such as the increase in family income. However, this does not suggest a real improvement in the family’s economic and financial conditions, but possibly reflects the increase in the number of people who started to contribute with some income because of the increase in the number of people who started to live in the same household. Despite the increase in family income during the pandemic, 61.5% of families earned up to 1 minimum wage (US$ 225.86) and the average number of people per household was 3 (*p* = 0.001), which is equivalent to an income of $ 2.5/day/person.

The addition of people in the same domestic space made the necessary isolation measures impossible at that time and provided a greater risk of contagion by COVID-19. Housing conditions are revealed to be one of the most important elements among the many competing factors for the greater or lesser spread of the SARS-CoV-2 coronavirus (29). It is known that in low-income areas, the sizes of rooms in the home are inadequate for the comfort and standard required for social isolation because of COVID-19. These houses do not meet the needs of families, especially those with more than three inhabitants (30).

On the other hand, as a protective factor for COVID-19, it was found that all evaluated individuals were vaccinated and two contracted the infection, but with good evolution (without gravity). Brazil has many social contrasts regarding access to basic sanitation, healthcare, transportation, education, and security, which reflects the contrasting human development indexes among different states and regions, and even among different areas within the same state or city. All these factors play a role in the risk of COVID-19 infection and dissemination, as well as in the ability of a given population to follow the isolation and social distancing measures (31).

During the pandemic, there was a discontinuity of access to the health services offered by SUS, as well as to the medications provided by this system. That way, for patients to be able to keep the continuous use drugs, the alternative was to acquire them by purchase or donation. Two important aspects must be considered during a pandemic: the confrontation of the new disease and the continuity of care for the set of health problems that continued to occur, overloading SUS which, prior to the pandemic, was already underfunded because of political decisions (32). Added to these factors of temporary deactivation of some health services, are the limited mobility of users to go in search of medicines and other healthcare services available. The current public health system in Brazil (SUS) has the principles of universality, scope and social participation. It is the main source of care for 75% of the population, reaching 87% in the northeast region. Health systems worldwide are experiencing profound and prolonged shocks from the COVID-19 pandemic. In Brazil, the acute shock caused by the COVID-19 resulted in a sharp drop in non-COVID healthcare procedures in the SUS (32). Bigoni et al. show that the distribution of resources did not prioritize the most vulnerable states, which were the most affected by the drop in procedures (32).

As for self-care, around 70% of those evaluated reported having maintained the daily hygiene of the limb affected by lymphedema. However, it was observed, perhaps caused by inefficient self-care, a higher presence of interdigital lesions, dermal ulcers, and number of ADLA crises, which justifies the increase in lymphedema volume and consequently, a greater risk of falling as evaluated by *TUG*. Repeated episodes of ADLA crises have been found to have a strong epidemiological association with the progression of lymphedema and are thought to be a major factor associated with disease advancement (33). As a possible factor for the increase in the volume of lymphedema, the suspension of physiotherapy to which patients were submitted in the pre-pandemic period is also added.

A significant proportion of the public health problem represented by lymphatic filariasis is due to impairment and disability related to lymphedema and hydrocele (4, 7, 34, 35). Therefore, the WHO indicates that national lymphatic filariasis programs must focus on MMPD. Important care recommendations include treating episodes of ADLA and preventing debilitating, painful episodes of ADLA and progression of lymphedema (36). Thus, the discontinuity of health services, greater difficulty in accessing drugs for continuous use and a greater number of ADLA episodes during the first year of the COVID-19 pandemic seem to have contributed to the worsening of lymphedema, greater risk of fallings, with an influence on the quality of life of residents with morbidity in areas that are endemic to lymphatic filariasis.

In assessing the quality of life of people with lymphedema coming from endemic areas of lymphatic Filariasis, several measurement tools have been used, in the form of questionnaires (37–44). In the present study, the *SF-36*, a generic questionnaire, was used, which was able to measure the clinical worsening of lymphedema in relation to bodily pain and social functioning of the patients.

In Sri Lanka, in the district of Colombo, the quality of life of patients with lymphedema was evaluated using two instruments (*SF-36* and the *30-item General Health Questionnaire-GHQ-30*). In the *SF-36* assessment, patients experienced worse physical functioning, greater limitations in physical health conditions, lower emotional wellbeing, worse social interaction, and more pain (45). The *SF-36* applied in our study was able to identify low scores in the eight domains analyzed, before and during the pandemic. On the other hand, we can observe that the scores in domains of limitations of physical health and emotional problems reflect slightly better in quality of life. The discontinuance of care for chronic conditions during the COVID-19 pandemic manifests itself in a disastrous way, as it involves the aggravation of chronic conditions such as in lymphatic filariasis. With the lack of assistance caused by access restrictions or people’s fear of seeking health services, chronic conditions tend to become unstable and increase in severity and cause deaths (46).

The findings obtained must be interpreted considering some limitations. First, the study described the outcome in the real-world setting, but without a control group, its effectiveness cannot be evaluated. Second, given the characteristics of the sample, prior and during the COVID-19 pandemic, it may show reduction in its representativeness, such as small number of participants, low male patients, an overall high level of literacy. These facts could not be controlled since the study model was to compare the impact before and during the pandemic among patients in follow-up with lymphedema, thus making it impossible to include new cases. Third, in obtaining information on hygiene maintenance, there may have been a memory bias, given that the data obtained in 2021 referred to the entire first year of the pandemic. This information was complemented with a detailed clinical examination of the affected limb.

Morbidity management and disability prevention remains a critically important aspect of the PGELF, particularly as countries approach validation of elimination of lymphatic filariasis as a public health problem, as is the case in Brazil. There is a need to use standardized instruments in the assessment of quality of life in the follow-ups of patients with morbidity and in the actions implemented in the PGELF. The worsening of the clinical status and quality of life in patients with filarial lymphedema during the COVID-19 pandemic with discontinuity in the assistance provided by the health system reiterates the importance of morbidity care both in individual care and in national programs.
